# Influence of Polymorphisms in the *HTR3A* and *HTR3B* Genes on Experimental Pain and the Effect of the 5-HT_3_ Antagonist Granisetron

**DOI:** 10.1371/journal.pone.0168703

**Published:** 2016-12-21

**Authors:** Sofia Louca Jounger, Nikolaos Christidis, Britt Hedenberg-Magnusson, Thomas List, Peter Svensson, Martin Schalling, Malin Ernberg

**Affiliations:** 1 Department of Dental Medicine, Section of Orofacial Pain and Jaw Function, Karolinska Institutet, and the Scandinavian Center for Orofacial Neurosciences (SCON), Huddinge, Huddinge, Sweden; 2 Department of Clinical Oral Physiology at the Eastman Institute, Stockholm, Public Dental Health, Stockholm, Sweden; 3 Department of Orofacial Pain and Jaw Function, Faculty of Odontology, Malmö University, and the Scandinavian Center for Orofacial Neurosciences (SCON), Malmö, Sweden; 4 Section of Orofacial Pain and Jaw Function, School of Dentistry, University of Aarhus, and the Scandinavian Center for Orofacial Neurosciences (SCON), Aarhus C, Denmark; 5 Center for Molecular Medicine, Karolinska Institutet, Solna, Stockholm, Stockholm, Sweden; University of Birmingham, UNITED KINGDOM

## Abstract

The aim of this study was to investigate experimentally if 5-HT_3_ single nucleotide polymorphisms (SNP) contribute to pain perception and efficacy of the 5-HT_3_-antagonist granisetron and sex differences. Sixty healthy participants were genotyped regarding *HTR3A (rs1062613)* and *HTR3B (rs1176744)*. First, pain was induced by bilateral hypertonic saline injections (HS, 5.5%, 0.2 mL) into the masseter muscles. Thirty min later the masseter muscle on one side was pretreated with 0.5 mL granisetron (1 mg/mL) and on the other side with 0.5 mL placebo (isotonic saline) followed by another HS injection (0.2 mL). Pain intensity, pain duration, pain area and pressure pain thresholds (PPTs) were assessed after each injection. HS evoked moderate pain, with higher intensity in the women (P = 0.023), but had no effect on PPTs. None of the SNPs influenced any pain variable in general, but compared to men, the pain area was larger in women carrying the C/C (*HTR3A*) (P = 0.015) and pain intensity higher in women with the A/C alleles (*HTR3B*) (P = 0.019). Pre-treatment with granisetron reduced pain intensity, duration and area to a lesser degree in women (P < 0.05), but the SNPs did not in general influence the efficacy of granisetron. Women carrying the C/T & T/T (*HTR3A*) genotype had less reduction of pain intensity (P = 0.041) and area (P = 0.005), and women with the C/C genotype (*HTR3B*) had less reduction of pain intensity (P = 0.030), duration (P = 0.030) and area compared to men (P = 0.017). In conclusion, SNPs did not influence experimental muscle pain or the effect of granisetron on pain variables in general, but there were some sex differences in pain variables that seem to be influenced by genotypes. However, due to the small sample size further research is needed before any firm conclusions can be drawn.

## Introduction

Pain is described as “an unpleasant sensory and emotional experience associated with actual or potential tissue damage, or described in terms of such damage” (International Association for the Study of Pain) [1]. One type of chronic pain condition in the orofacial area is temporomandibular disorder (TMD). It affects approximately 10–15% of the adult population [2, 3] and is twice as common in women as men [4, 5]. The most common type of TMD is myalgia of the jaw muscles. The symptoms include restricted mouth opening, pain upon chewing and function, muscle soreness, pain refal and headache which leads to a decreased quality of life [6, 7].

The mechanisms that underlie chronic pain conditions such as TMD myalgia and their sex differences are poorly understood. However, results indicate that release of inflammatory substances, such as serotonin (5-HT) may be involved at a peripheral level. 5-HT is a monoamine that derives from tryptophan and serves as a neurotransmitter. 5-HT is found both in the peripheral nervous system (PNS) and in the central nervous system (CNS) and participates in pain amplification as well as in pain inhibition in the serotonergic system depending which receptor subtype it activates [8]. In the PNS, 5-HT participates in pain mediation via 5-HT_3_ receptors and may be released by trauma or ischemia [9, 10]. Previous studies have reported increased masseter muscle levels of 5-HT in patients with chronic myalgia and a positive correlation to muscle pain and tenderness [11, 12]. Other studies have also reported higher intramuscular 5-HT levels in trapezius muscle of patients with chronic trapezius myalgia and fibromyalgia [13–16]. Experimental studies have shown that local administration of drugs that block 5-HT_3_ receptors, such as granisteron, have a pain reducing analgesic effect and increase the pressure pain threshold (PPT) in TMD myalgia [17] as well as after experimentally induced pain in the masseter muscle in healthy subjects [18, 19]. However, there are sex differences reported in response to 5-HT_3_ blockers. One study reports increased PPTs in women but not in men, after intramuscular co-injection of granisetron and 5-HT into the masseter muscle [19]. On the contrary, two other studies reported increased PPT only in the men, both after oral and local administration of granisetron [18, 20].

Clinical studies have also shown that 5-HT_3_ antagonists are effective analgesics for various pain conditions, such as TMD myalgia, localized trapezius myalgia and fibromyalgia [21–24]. However, in most clinical trials a great proportion of the patients, regardless of diagnosis, do not respond to 5-HT_3_ antagonists. This finding, and the sex differences in response to granisetron might hypothetically be due to differences in genotypes of the 5-HT_3_ receptors.

The 5-HT_3_ receptor is a ligand-gated ion channel that has five subunits, 5HT3A-E, coded by the genes *HTR3A-E*. The *HTR3A-B* genes are positioned on chromosome 11q23.1—q23.2 and contain 7 and 9 exons, respectively. The *HTR3C-E* genes are positioned on chromosome 3q27.1. The 5-HT3A subunit form functional homomeric 5-HT_3A_ receptors, while the other subunits cannot assembly into functional receptors alone. Instead they form heteromeric receptors with the 5-HT_3A_ receptor. These exhibit quantitatively different functional properties compared with homomeric 5-HT_3A_ receptors. For example, the 5-HT_3A/B_ complex has a 40-fold increased single-channel conductance compared to homomeric 5-HT_3A_ receptors [25]. Interestingly 5-HT_3_ receptors with a single nucleotid polymorphism (SNP) 386A>C (rs1176744) in the *HTR3B* gene, resulting in a substitution of tyrosine to serine (Y129S), showed increased response to 5-HT compared to receptors with wild type tyr129 [26]. This could indicate that subjects carrying the C-allele of this SNP may be more sensitive to pain perception.

While several studies have related polymorphisms in the *HTR3A* and *HTR3B* genes to psychiatric disorders [27], only a few studies have shown an association to chronic pain. One study investigated sequence variation in the *HTR3A* and *HTR3B* gene polymorphisms in fibromyalgia patients in order to reveal a possible involvement in its pathophysiology. No involvement was proved, but a novel genetic variant in the *HTR3A* gene, 97G>A (Ala33Thr) was identified in two patients [28]. A SNP in the *HTR3A* gene, c.-42C>T (C178T; rs1062613) was reported more frequent in patients with irritable bowel syndrome (IBS) compared to healthy controls [29]. In another study the same SNP was associated with amygdala responsiveness, with patients carrying the C/C genotype showing greater symptom ratings and anxiety than patients carrying the T-allele [30]. In addition, the SNP showed an involvement in the etiology of eating disorders in humans [31] and an association with bipolar disorder [29, 32]. With respect to the *HTR3B* gene, the SNP (rs1176744) was correlated to major depression in Japanese women [33] and to bipolar disorders [34]. Subjects carrying the C genotype of this SNP were also associated to higher scores on the Pain Catastrophizing Scale (PCS), suggesting a role of 5-HT pathways in pain related catastrophizing [35].

Moreover, *HTR3A* and *HTR3B* polymorphisms have also been shown to serve as predictors for the effectiveness of 5-HT_3_ antagonists [27] as well as the antidepressant response to the serotonin selective reuptake inhibitor paroxetine [36]. Taken together these findings indicate that genetic variants in the *HTR3A* and *HTR3B* may be involved in the pathophysiology of depressive disorders and the clinical effect of 5-HT_3_ antagonists.

Depression and pain share similar pathways [37]. Thus, polymorphisms in the serotonergic system may also be involved in the pathophysiology of chronic muscle pain. However, the role of 5-HT polymorphisms in pain mediation and the efficacy of 5-HT_3_ antagonists in orofacial muscle pain have not yet been explored. The aim of this study was therefore to investigate if the *HTR3A* (rs1062613) and *HTR3B* (rs1176744) SNPs contribute to pain perception and the analgesic efficacy of the 5-HT_3_ antagonist granisetron in an experimental human muscle pain model. A second aim was to investigate if specific *HTR3AB* genotypes could explain any sex differences in pain responses and analgesic effects of granisetron in this experimental model.

## Materials and Methods

The study is partly built on experimental data from a previous study from this group in which the analgesic effect of granisetron on hypertonic-induced masseter muscle pain was investigated [18], but with the addition of more participants to be able to study the genetic influences of the selected *HTR3A* and *HTR3B* SNPs. The participants in the previous study (n = 30) were therefore contacted and asked to donate a saliva or blood sample for genetic analyses. From 17 of them saliva or blood could be obtained.

The project followed the guidelines according to the Declaration of Helsinki as well as Good Clinical Practice and was approved by the Regional Ethical Review Board in Stockholm, Sweden (2011/1955-31/2) and by the Medical Products Agency in Uppsala, Sweden (2011-006206-27, Dnr 151:2011/96710). All participants received written and verbal information of the study before participating and gave their written consent.

### Participants

Sixty healthy participants, 30 men and 30 aged-matched women with a mean (SD) age of 26.8 (3.9) years participated in the study. The participants were recruited by advertisement and from colleagues and students at the Department of Dental Medicine at the Karolinska Institutet, Huddinge, Sweden, where the study was conducted.

A power calculation showed that 60 participants would be sufficient to detect a mean (SD) difference in pain intensity between alleles of 10 (10) % with 80% power and a significance level of 5%.

Inclusion criteria were age over 18 years and good general health. Exclusion criteria were any pain-related TMD diagnosis or other orofacial pains, diagnosed systemic muscular or joint diseases (e.g. fibromyalgia and rheumatoid arthritis), whiplash-associated disorder, neuropathic pain or neurological disorders, pregnancy or lactation, and high blood pressure.

### Experimental protocol

The study used a randomized, placebo-controlled and double-blinded design with one session that lasted for 1.5 hours. The study followed exactly the same protocol as in the previous study [18].

Briefly, the participant was seated in a conventional dental chair and were first screened with a clinical examination according to the Research Diagnostic Criteria for TMD (RDC/TMD) [38] in order to establish that they did not have any pain-related TMD. If the participant was included in the study, venous blood or saliva was sampled for genetic analyses. Baseline assessments of pain levels (0 according to inclusion criteria) and PPTs were then performed. The participants hereafter received bilateral injections of hypertonic saline (HS) into the masseter muscles to evoke pain. This was done as an internal control to reassure that similar pain was induced on both sides [18], but also to investigate the influence of *HTR3A* and *HTR3B* polymorphisms on pain variables. After at least 30 min, when pain from the HS injection since long had subsided, the masseter muscle was pre-treated with granisetron on one side and placebo on the contralateral side followed by another bilateral HS injections 2 min later. The sides for granisetron and placebo injection were randomized by a random number generator (www.randomization.com), such that half of the participants received granisetron on the right side, and the other half on the left side. The injections were given in a double-blinded manner. After each HS injection pain intensity, pain duration, pain area and PPTs were assessed, in accordance to previous study [18].

### Injections

Intramuscular injections of HS is a valid and commonly used experimental pain model mimicking some aspects of chronic local myalgia [39]. It has an acute pain character with a sensation of deep, diffuse pain and pain spread or refal and usually lasts less than 10 minutes. A total of 0.2 mL sterile HS (58.5 mg/mL) was used to induce pain. The HS was injected into the relaxed muscle during 10 s with the aid of an infusion pump (Harvard Infusion Pump 22, Harvard Apparatus, Great Britain) to ensure that the same infusion rate (0.02 mL/s) was used for all participants.

Granisetron is a specific 5-HT_3_ antagonist used to treat chemotherapy-induced nausea and vomiting. It is considered to be a safe and well-tolerated drug with few side effects [40]. Granisetron has a long half-life since it is metabolized in the liver. A total of 0.5 mL of granisetron (Gra; Kytril^®^, 1 mg/mL, Roche, Stockholm, Sweden) and placebo (isotonic saline, 9 mg/mL) was used as in previous studies from this group [24, 41, 42]. The same infusion rate as for the HS injections (0.02 mL/s) was used, i.e. the injections were performed over 25 seconds.

**Fig 1:**
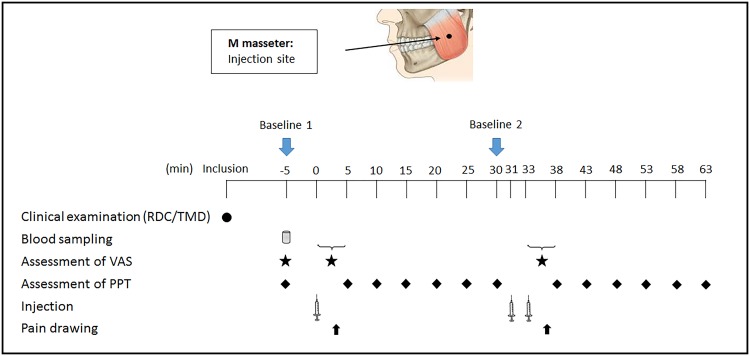
Flow chart of the experimental setting. The figure shows the time points (minutes) for the clinical examination, blood sampling, assessment of pain intensity (VAS), pressure pain thresholds (PPT), time points for injections and pain drawings. At the time point 0 minutes, bilateral injections of hypertonic saline (0.2 mL) into the masseter muscles were performed. At the time point 31 minutes, pre-treatment with granisetron (0.5 mL) on one side of the masseter muscle and placebo (0.5 mL) on the other side was done. Two minutes later, a second bilateral injection of hypertonic saline (0.2 mL) was performed. Pain intensity, pain duration and pain area were assessed after each injection.

The injections were done in the relaxed muscle using a 2-mL syringe with a 19-mm needle (diameter 0.4 mm) inserted into the masseter muscle to a depth of approximately 15 mm. The most prominent point of the masseter muscles was used. The points were determined by asking the participant to clench their teeth while the muscle belly was palpated. Roughly this point is located in the center of the muscle in the anterior-posterior direction and approximately 2 cm superior to the lower border of the mandible. No skin surface anesthesia was used before the insertion. To ensure that the granisetron/placebo injection and the subsequent HS injection targeted exactly the same site, the same needle was kept in the muscle between injections, and only the syringes were changed (Fig 1).

### Assessments of pain

Pain was assessed with a 0–100 mm visual analogue scale (VAS) marked with the end points “no pain” and “the worst pain experienced”. Immediately after the injections the participants were instructed to mark their pain level on the scale every 15th second until pain had subsided with a maximum duration of 300 s. Thereafter the participants were asked to recall the maximum pain spread or referral during the HS injection and to mark this on a face chart, depicting the lateral side of the head.

### Assessments of pressure pain threshold

The PPT was recorded with an electronic algometer (Somedic Sales AB, Höör, Sweden) on the most prominent point of the masseter muscles (injection sites). The algometer consists of a 1-cm^2^ probe and the tip of the algometer is covered with a 1-mm-thick rubber pad. A standardized pressure rate of 50 kPa per second was used. The participants were instructed to press a signal button as soon as the pressure turned into pain. At baseline three measurements with an interval of 2 minutes were taken bilaterally, and the mean value was used in the analyses. After each HS injection, the PPTs were recorded twice every 5 min for 30 min in the same muscle to reduce the risk of sensitization by repeated pressure stimuli [18, 42] (Fig 1). The mean value of the two measurements was used in the analyses.

### DNA analysis

Whole blood (4 mL) was collected from a peripheral vein using 4 ml Vacutainer tubes containing an ethylenediaminetetracetic acid (EDTA) solution. If blood could not to be drawn due to technical reasons, saliva was instead sampled using OG-500 kits (DNA Genotek Inc, Ontario, Canada). DNA was extracted from blood or saliva using standard methods. The SNPs rs1062613 and rs1176744 from the *HTR3A* and *HTR3B* genes were genotyped on the Applied Biosystems Quantstudio 7 Flex Real-Time PCR System from Thermo Fischer Scientific, Carlsbad, CA by using allele specific Taqman MGB probes labeled with fluorescent dyes FAM and VIC, according to the manufacturer´s protocol. This was done according to previous studies [43–45].

### Statistics

Data were analyzed with SigmaPlot for Windows, version 11 (Systat Software Inc., Chicago, IL, USA). The Shapiro-Wilk’s test was used to evaluate if data was normally distributed. As most data were not normally distributed and/or ordinal non-parametric statistics were used. Descriptive data are presented as number of participants (n), frequencies (%), and median with interquartile range (IQR) depending on type of data.

From the data of VAS pain intensity after injections, the maximum individual pain level (peak pain; 0–100) as well as the total pain duration (s) was used for statistical analyses. The pain drawings were analyzed using a transparency with 1.5–1.5 mm squares placed over the pain drawings and the squares were counted. Squares partly inside the border were added up to full squares. The area was expressed in arbitrary units (au) [18, 42]. For PPT, the values obtained after HS injections (5–30 min) were normalized (percent change) to the baseline value.

For analyses of granisetron effects on HS-induced peak pain, pain duration and pain area, the values obtained after granisetron injection were normalized to the values obtained after the first HS injection. To compare the effect of different SNPs on changes in PPTs, the average of the values obtained at the different time points (5, 10, 15, 20, 25 and 30 min) was calculated (PPT_mean_). This value was then normalized to the baseline value and used in the analyses.

Wilcoxon test was used to analyze differences in pain variables and PPT after injections while Mann-Whitney U-test was used to analyze sex differences. The effect of the different SNPs on pain variables and PPTs after HS injection and granisetron, ANOVA on ranks (Kruskall-Wallis test) or Mann-Whitney U-test was used. The level of significance was set to P < 0.05.

## Results

None of the participants dropped out. The results regarding HS effects and analgesic effects of granisetron on pain variables and PPT did not differ from those of our previous study [18], why these are only briefly presented.

### SNPs

The frequencies of the genotypes are shown in Table 1. The most common genotype in the *HTR3A* rs1062613 was C/C followed by C/T and T/T. Since only two participants were carrying the homozygous genotype T/T in the *HTR3A* gene, they were combined with the heterozygous C/T genotype and compared to the homozygous C/C genotype. In the *HTR3B* rs1176744, the most common genotype was A/C followed by A/A and C/C. There were no sex differences in allele distribution for any of the polymorphisms.

**Table 1:** The distribution of the 5-HT_3_-polymorphisms *HTR3A* (rs1062613) and *HTR3B* (rs1176744).

	*HTR3A*			*HTR3B*		
	C/C	C/T	T/T	A/A	A/C	C/C
Men (n = 30)	16 (27%)	12 (20%)	2 (3%)	10 (17%)	15 (25%)	5 (8%)
Women (n = 30)	20 (33%)	10 (17%)	0 (0%)	13 (22%)	11 (18%)	6 (10%)

There were no sex differences in allele distributions for any of the 5-HT_3_ polymorphisms.

### Effect of gene variants on experimentally induced muscle pain

**Fig 2:**
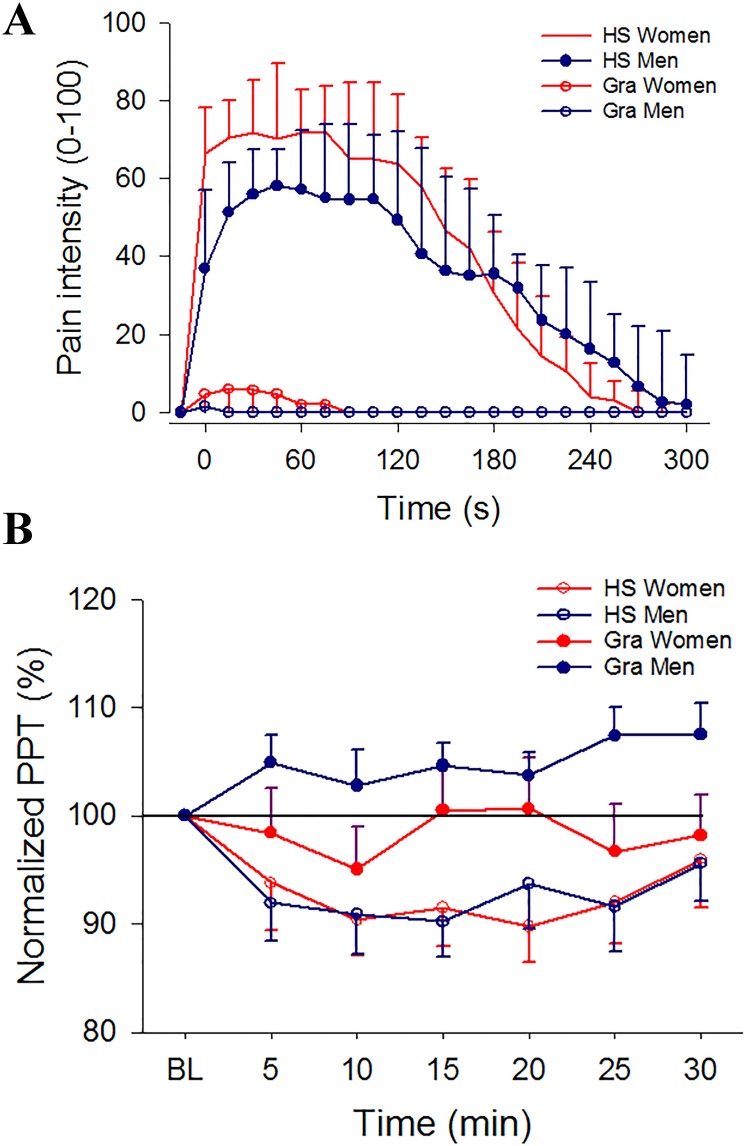
**(A). Pain intensity, pain duration after HS injection alone and after pre-treatment with granisetron**. The figure shows pain intensity and pain duration after hypertonic saline (HS) injection alone or after pre-treatment with granisetron (Gra) in 60 healthy participants (30 women and 30 age-matched men). HS evoked pain with higher intensity in the women (P = 0.023). Pre-administration of Gra before HS-injection significantly reduced pain intensity and duration (P < 0.001), with less pronounced effect in women compared to men (P = 0.035 and P = 0.016, respectively). **(B). Pressure pain thresholds after HS injection alone and after pre-treatment with granisetron**. The figure shows pressure pain thresholds (PPT) after hypertonic saline (HS) injection alone or after pre-treatment with granisetron (Gra) in 60 healthy participants (30 women and 30 age-matched men). PPTs were normalized to baseline. There were no significant changes in PPTs after injection of HS. Pre-administration of Gra before HS-injection had no significant effect on PPTs.

**Fig 3:**
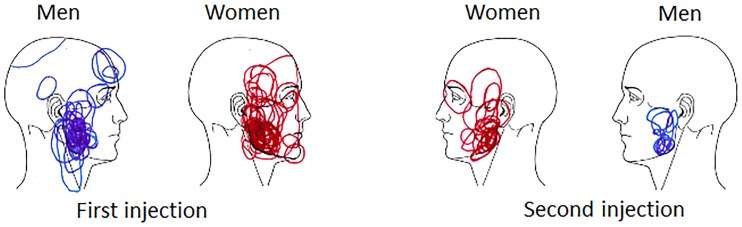
Pain distribution in the masseter muscle after HS injection alone and after pre-treatment with granisetron. The figures show the pain distribution in the masseter muscle after injections of hypertonic saline (HS) alone (first injection) and after pre-treatment with granisetron (0.5 mL) and placebo (0.5 mL isotonic saline) followed by a second injection of hypertonic saline (second injection) in 60 healthy participants (30 women and 30 age-matched men). The pain area after HS injection did not differ significantly between sexes. The pain area was significantly smaller after pre-treatment with granisetron (P < 0.001) with greater reduction in men than women (P = 0.007).

HS injection into the masseter muscle induced pain of moderate intensity that lasted approximately 4 min with higher peak pain intensity in women (Fig 2). The pain area was mostly localized to the masseter muscle but some participants reported pain referral to the temporalis, teeth and forehead. There were no significant sex difference in pain area (Fig 3). There were no differences in pain intensity, pain duration or pain area between the different *HTR3* alleles. Women with the C/C type of the *HTR3A* gene had larger pain area than men, and women with the A/C type of the *HTR3B* gene reported higher pain intensity than men (Table 2). None of the other alleles showed any sex differences in pain intensity, pain duration or pain area.

**Table 2:** Pain variables after injection of hypertonic saline.

	*HTR3A*		*P*	*HTR3B*			*P*
	C/C	C/T & T/T		A/A	A/C	C/C	
**Pain intensity**							
Women	77 (31)	76 (25)	*0*.*775*	74 (27)	85 (22)	58 (29)	*0*.*284*
Men	61 (38)	64 (30)	*0*.*672*	67 (36)	64 (32)	52 (16)	*0*.*137*
***P***	*0*.*092*	*0*.*114*		*0*.*877*	***0*.*019***	*0*.*633*	
**Pain duration**							
Women	255 (53)	263 (41)	*0*.*462*	255 (30)	255 (68)	233 (38)	*0*.*510*
Men	240 (109)	300 (56)	*0*.*175*	300 (45)	285 (90)	180 (165)	*0*.*418*
***P***	*0*.*833*	*0*.*199*		*0*.*359*	*0*.*979*	*0*.*725*	
**Pain area**							
Women	54 (92)	48 (33)	*0*.*860*	42 (40)	54 (102)	68 (83)	*0*.*637*
Men	20 (15)	47 (68)	*0*.*081*	25 (53)	24 (33)	18 (30)	*0*.*137*
***P***	***0*.*015***	*0*.*907*		*0*.*352*	*0*.*253*	*0*.*126*	
**PPT after injection**							
Women	96 (18)	95 (15)	*0*.*912*	95 (16)	93 (13)	112 (24)	*0*.*057*
Men	92 (19)	85 (21)	*0*.*603*	87 (17)	97 (18)	81 (21)	*0*.*741*
***P***	*0*.*738*	*0*.*661*		*0*.*780*	*0*.*917*	*0*.*126*	

The median (IQR) peak pain intensity (0–100 mm VAS), pain duration (s), pain area (au) and pressure pain thresholds (PPT) normalized to baseline (% of values) are shown. The participants are divided into groups depending on allele distribution in the *HTR3A* and *HTR3B* genes.

IQR = interquartile range (75 percentile minus 25 percentile). Bold figures denote significant differences (P < 0.05).

### Effect of gene variants on granisetron effects

After pre-treatment with granisetron, the subsequent HS injection induced pain with lower pain intensity, shorter duration and smaller pain area than HS alone. The reductions of pain intensity, duration and area were less pronounced in women (Figs 2 and 3). There were no differences between the *HTR3A* or *HTR3B* genotypes in treatment effect on pain intensity, pain duration or pain area for the group as a whole. Women carrying the T allele of the *HTR3A* gene had less reduction of pain intensity and pain area than men with this genotype, and women homozygous for the C allele of the *HTR3B* gene had less reduction of pain intensity, pain duration and pain area than men (Table 3).

**Table 3:** Pain variables after pretreatment with granisetron after the second injection of hypertonic saline.

	*HTR3A*		*p*	*HTR3B*			*p*
	C/C	C/T & T/T		A/A	A/C	C/C	
**Pain intensity**							
Women	12 (57)	41 (59)	*0*.*401*	9 (46)	15 (58)	59 (59)	*0*.*464*
Men	3 (44)	10 (19)	*0*.*948*	10 (18)	3 (52)	0 (3)	*0*.*433*
***p***	*0*.*270*	***0*.*041***		*0*.*436*	*0*,*521*	***0*.*030***	
**Pain duration**							
Women	28 (57)	35 (33)	*0*.*965*	50 (58)	32 (42)	26 (21)	*0*.*804*
Men	0 (43)	5 (34)	*0*.*718*	29 (44)	0 (33)	0 (0)	*0*.*249*
***p***	*0*.*099*	*0*.*070*		*0*.*254*	*0*.*186*	***0*.*030***	
**Pain area**							
Women	9 (82)	20 (50)	*0*.*810*	10 (41)	10 (83)	15 (55)	*0*.*848*
Men	0 (31)	0 (0)	*0*.*364*	0 (8)	0 25)	0 (0)	*0*.*321*
***p***	*0*.*228*	***0*.*005***		*0*.*245*	*0*.*166*	***0*.*017***	
**PPT after injection**							
Women	103 (17)	100 (26)	*0*.*708*	96 (23)	107 (17)	102 (25)	*0*.*149*
Men	105 (12)	106 (11)	*0*.*724*	105 (15)	106 (12)	104 (5)	*0*.*924*
***p***	*0*.*454*	*0*.*230*		*0*.*100*	*0*.*716*	*0*.*662*	

**The** median (IQR) peak pain intensity (0–100 mm VAS), pain duration (s), pain area (au) and pressure pain thresholds (PPT) normalized to baseline 2 (% of values) is shown. The participants are divided into groups depending on allele distribution in the *HTR3A* and *HTR3B* genes. IQR = interquartile range (75 percentile minus 25 percentile). Bold figures denote significant differences (P < 0.05).

PPT did not change significantly after pre-treatment with granisetron (Fig 2). Neither were there any differences in PPT_mean_ between the polymorphisms in the *HTR3A* and *HTR3B* genes on the effect of granisetron for the entire group (Table 3).

## Discussion

The main results of this study show that the rs1062613 and rs1176744 polymorphisms in the *HTR3A* and *HTR3B* genes, respectively did not seem to directly affect HS-induced muscle pain, or the significant analgesic effect of granisetron. Yet, there were some indications of minor sex differences in the effect on pain variables between the different genotypes.

### Influence of *HTR3* polymorphisms on pain variables and the efficacy of granisetron

Although several studies have reported an involvement of polymorphisms in the *HTR3A* (rs1062613) and *HTR3B* (rs1176744) genes and psychiatric conditions, such as eating [31] and bipolar disorders [32], relatively few have explored their role in pain conditions. Two studies have examined the influence of the *HTR3A* (rs1062613) polymorphism in patients with IBS. The first study reported that using a minor allele dominant model this polymorphism was more frequent in IBS patients (n = 100) than controls (n = 100) [29]. Another study (26 women with IBS, 29 controls) reported higher severity of gastrointestinal symptoms and greater amygdala responses to both emotional and non-emotional stimuli in IBS patients homozygous for the C allele compared to T carriers. Importantly though, there was no association to abdominal pain ratings [30]. The latter result is in line with the findings of no differences in experimental pain variables or PPT between carriers of the different allele types of the *HTR3A* (rs1062613) polymorphism in this study. With respect to the *HTR3B* gene polymorphism (rs1176744) to our knowledge, only one pain study has been conducted in healthy participants. In that study the G allele (i.e. C allele) was negatively correlated to PCS scores [35]. This seemingly contrasts the results of the present study of no association between carriers of the different *HTR3B* genotypes. However, the results cannot be directly compared since pain was not actually evoked in their study.

Neither was there any influence of *HTR3A* and *HTR3B* genotypes on the efficacy of granisetron for the group as a whole. There are no studies that have investigated the influence of *HTR3* polymorphisms on the analgesic effect of 5-HT_3_ antagonists, but a few studies have investigated the influence on chemotherapy-induced nausea and vomiting. One study identified 21 polymorphisms in the *HTR3A* gene including rs1062613 in 233 cancer patients, but found no association to nausea or vomiting [46]. The same authors also investigated the influence of polymorphisms in the *HTR3B* gene on the antiemetic effect of 5-HT_3_ antagonists. Of 13 identified polymorphisms they found that a 3–base pair deletion variant in the promoter region (−100_−120delAAG deletion) was associated with higher frequency of vomiting. There was also a trend to a lower number of vomiting episodes for patients with the rs1176744 polymorphism, but that was linked to the −100_−120delAAG deletion [47]. Another recent study found no association between the rs1176744 polymorphism and the effect of ondansetron for post-operative vomiting [48]. Overall, the present findings show no direct association between the *HTR3AB* polymorphisms (rs1062613, rs1176744) and experimental muscle pain by HS or the effect of granisetron.

### Sex differences and experimental muscle pain

As in a previous study from our research group [18] there were sex differences in pain evoked by HS with women in general reporting higher pain intensity than men. Interestingly, this sex difference may be influenced by allele variants as the injection induced larger pain area only in women carrying the *HTR3A* C/C genotype. Similarly, only women with the A/C genotype in the *HTR3B* gene reported higher pain intensity compared to men. In a previous study the C/C genotype in the *HTR3A* gene was associated with increased anxiety and amygdala responsiveness during emotional and non-emotional tasks in patients with IBS as well as in healthy controls [30]. In another study, subjects carrying the C-allele in the *HTR3B* gene *HTR3B* may be more sensitive to pain perception since the substitution of tyrosine to serine (Y129S), showed increased response to 5-HT compared to receptors with wild type tyr129 [26]. Even though the results of the present study should be interpreted with caution with respect due to the small number of participants carrying the various SNPs, one cannot exclude the possibility that women carrying the C-allele of both the *HTR3A* and *HTR3B* gene could be at higher risk both for increased anxiety and pain, since pain and depression share similar pathways [37].

Also, there were sex differences regarding the efficacy of granisetron. Women carrying the T allele in the *HTR3A* gene had less reduction of pain intensity and pain area than men, and women homozygous with the C allele of the *HTR3B* gene had less reduction of pain intensity, pain duration and pain area than men. Interestingly, several studies have described a sex difference in pain response to analgesics such as opioids were women seem to respond better than men [49–51]. One study showed that women had a better analgesic effect of opioids, after they underwent surgery for the removal of the third molar teeth than men [51]. Genetic influences are one of multiple mechanisms thought to be involved and may explain the sex differences in opioid analgesia [49]. With this in mind, the sex differences in response to granisetron and other analgesics may be due to the genetic variances, and possibly women homozygous for the C allele of the *HTR3B* gene may respond better to analgesics.

In line with previous studies the HS injection did not influence PPTs, giving further evidence that HS does not sensitize nociceptors [18]. However, women had lower PPTs both at baseline and after HS injection. The PPTs on the other hand, were not influenced by gene variants for any SNP. However, several different 5-HT polymorphic variants interact which complicates the picture. Both the rs1062613 and rs1176744 have been shown to interact with most of the other polymorphisms in their respective genes [46, 47]. Further, the mechanisms behind chronic pain are multifactorial and therefore SNPs occurring in different genes of the same metabolic pathway may interact in combination, intensifying the effect. A previous study highlighted different effects of SNPs when combined together. A combination of six SNPs from the genes involved in the 5-HT-receptor pathways was associated with greater odds of localized TMD [52]. In addition, the groups became significantly smaller when comparing sex differences in each genotype. Therefore the power of the analyses was probably too low. Also, several other factors are involved in pain perception such as ethnic and cultural experiences [53]. Thus our results must be interpreted with caution. Further studies are needed to further explore associations between *HTR3* genes, biological sex and pain as well as the analgesic effect of granisetron.

### Study limitations

There are some limitations in this study that needs to be addressed. Since there was no previous article to base a power calculation on, the relative small sample size limited the power of the statistical analyses. A larger population would therefore be preferable in future studies. Also, the genotyping was performed after the experiment. This resulted in uneven sized groups of genotypes, for example only two participants with the *HTR3A* rs1062613 carried the T/T genotype, which complicated comparisons between groups. To prevent this in future studies, genotyping of the participants should preferably be performed prior to the experiment [35]. When performing the statistical analyses, participants carrying the T/T genotype were combined with the group having the C/T genotype and compared to the homozygous C/C group, in line with previous studies [30]. Nevertheless, the results did not even show any tendencies to differences in pain variables or efficacy of granisetron between the different gene variants. Further, other polymorphisms in the *HTR3* genes could be investigated. For example the -100-102AAG deletion polymorphisms of the HT3B gene, which has been reported to influence the efficacy of ondansetron in preventing chemotherapy-induced and post-operative emesis [47, 48]. Finally, an experimental pain model that also induces mechanical sensitization, e.g. glutamate injection [54, 55] might better capture the effect of granisetron on PPT changes.

Another area of interest, are questionnaires of psychological nature such as the Perceived Stress Scale that measures the perception of stress, the State and Trait Anxiety Inventory (STAI) that is an instrument used to measure anxiety in adults and the PCS which measures thoughts and feelings when experiencing pain. Such questionnaires could have been useful tools to assess if there were differences also in stress, anxiety or depression levels between the participants that were associated to a specific allele or genotype. Also, in future studies multiple 5-HT polymorphisms could be investigated, both independently and combined, in order to learn more about the pathophysiology of chronic pain disorders. This might ultimately lead to different treatment approaches.

Repeated painful stimuli are known to activate the diffuse noxious inhibitory control (DNIC) which lead to less pain. In this study two repeated injections of hypertonic saline were given which may have activated DNIC. However, previous study with repeated injections of hypertonic saline in the trapezius muscle, with 15 minutes apart, showed no differences in pain [56]. This shows that any possible DNIC effect was not measurable, and therefore could not influence the outcome of our results.

## Conclusions

This study showed that the *HTR3A* rs1062613 and *HTR3B* rs1176744 polymorphisms do not seem to directly influence experimental muscle pain in healthy individuals. However, women reported higher pain intensity and larger pain area than men, which might partly be attributed to genotype. Also the difference in granisetron effect on pain variables may be attributed to these polymorphisms to some extent. It is clear that the serotonergic system is involved and associated with the pathways of pain. Therefore, the possibility that 5-HT polymorphisms could influence, predict or be a risk factor in developing pain disorders such as chronic myalgia cannot be excluded. However, further studies are warranted in order to systematically explore several 5-HT polymorphisms to be able to draw any further conclusions.

## Supporting Information

S1 Appendix Data set showing pain over time. (PDF)

S2 Appendix Data set showing pain variables. (PDF)

S3 Appendix Data set showing pain area. (PDF)

S4 Appendix Data set showing PPT over time. (PDF)

S5 Appendix Approval from the Ethical Review Board. (PDF)

S6 Appendix Approval from the Medical Products Agency. (PDF)
