# Supplementary material for: Influence of Polymorphisms in the HTR3A and HTR3B Genes on Experimental Pain and the Effect of the 5-HT3 Antagonist Granisetron
Source: PLoS One. 2016 Dec 21;11(12):e0168703. doi: 10.1371/journal.pone.0168703 (PMC5176308; doi:10.1371/journal.pone.0168703)
Supplement: S1 Appendix — (PDF) [file pone.0168703.s001.pdf]

Pat nr	BL	Hyp-Gra 0	Hyp-Gra 15	Hyp-Gra 30	Hyp-Gra 45	Hyp-Gra 60	Hyp-Gra 75	Hyp-Gra 90	Hyp-Gra 105	Hyp-Gra 120	Hyp-Gra 135	Hyp-Gra 150	Hyp-Gra 165	Hyp-Gra 180	Hyp-Gra 195	Hyp-Gra 210	Hyp-Gra 225	Hyp-Gra 240
1	0	71	82	84	87	87	87	87	88	84	67	47	38	27	16	11	10	8
2	0	78	84	89	93	95	97	97	94	90	79	71	54	42	33	26	17	0
3	0	20	25	29	36	36	39	39	39	39	37	37	37	33	24	17	7	0
4	0	61	62	61	52	62	63	64	64	64	55	53	50	36	24	10	7	4
5	0	92	92	96	93	80	80	72	72	70	60	52	47	16	7	3	1	0
9	0	37	43	52	43	34	22	12	3	0	0	0	0	0	0	0	0	0
11	0	6	7	7	7	7	7	8	9	8	7	6	6	6	7	7	6	6
13	0	95	92	82	81	81	68	47	11	3	2	2	7	4	3	2	0	0
14	0	72	73	73	63	52	47	40	37	35	27	21	25	22	19	16	12	9
15	0	98	98	99	99	97	99	100	100	100	65	50	50	25	15	3	3	0
16	0	45	50	53	54	57	54	51	47	27	7	0	0	0	0	0	0	0
19	0	20	25	32	38	50	50	55	55	54	35	28	13	2	0	0	0	0
23	0	72	72	72	72	70	70	68	63	59	29	25	18	10	2	3	1	0
25	0	17	25	29	36	36	36	34	31	30	18	17	10	3	0	0	0	0
26	0	12	26	27	20	16	14	13	7	5	2	1	1	1	0	0	0	0
27	0	49	54	57	57	54	53	47	41	40	22	15	6	2	1	0	0	0
29	0	11	14	17	18	20	21	23	25	26	19	18	16	17	16	15	14	12
31	0	52	61	65	65	65	65	63	47	38	27	23	19	15	9	5	2	2
32	0	35	56	66	70	74	76	76	76	69	52	36	12	4	2	0	0	0
33	0	48	50	47	45	39	39	34	27	31	22	24	17	14	11	7	2	0
35	0	6	18	34	42	45	45	46	50	48	45	40	37	34	32	28	24	16
36	0	0	61	87	92	96	96	95	94	88	87	86	84	71	53	38	39	29
37	0	70	67	65	58	52	47	44	40	35	34	22	16	5	2	0	0	0
38	0	19	23	36	36	46	46	60	55	56	41	34	34	25	20	16	12	7
39	0	38	41	41	40	39	37	35	32	29	29	26	15	11	7	3	1	1
40	0	52	55	57	64	68	67	69	69	71	66	52	46	37	28	10	3	0
41	0	50	80	81	80	82	84	85	82	71	60	58	50	49	48	34	33	33
42	0	63	75	86	86	90	90	93	93	93	91	90	66	66	55	48	48	33
43	0	10	30	32	32	30	25	24	22	19	12	10	10	2	0	0	0	0
44	0	80	80	79	77	76	76	60	49	47	36	30	30	19	10	0	0	0
45	0	10	18	10	10	9	9	9	9	9	8	8	3	0	0	0	0	0
46	0	90	92	90	87	85	84	81	76	75	67	62	56	48	45	38	27	15
47	0	18	33	62	63	63	63	59	59	57	58	54	47	15	13	9	7	5
48	0	73	84	91	93	96	95	97	98	98	97	97	98	95	95	92	88	73
49	0	57	59	64	67	69	73	73	73	70	69	68	66	30	22	18	19	8
50	0	92	81	95	85	81	81	81	79	73	74	69	67	60	41	22	5	2
51	0	11	18	18	18	18	15	15	15	9	8	0	0	0	0	0	0	0
52	0	96	96	97	100	100	100	100	100	100	97	90	85	84	84	85	73	51
53	0	55	59	63	63	64	65	65	66	66	64	63	63	60	59	48	30	18
54	0	79	64	78	89	70	71	71	70	71	71	77	77	77	68	63	54	43
55	0	37	57	75	75	75	68	73	69	68	52	52	41	26	26	12	12	12
56	0	55	49	49	49	49	49	49	52	51	67	66	66	60	48	44	41	40
57	0	32	65	68	69	69	69	69	66	59	73	66	58	53	35	35	15	11
58	0	74	80	77	77	75	76	75	70	68	53	53	53	44	37	31	30	30
59	0	19	25	29	29	29	29	27	26	27	16	12	5	3	0	0	0	0
60	0	7	12	30	43	69	69	87	84	86	74	49	47	45	44	41	37	31
61	0	76	76	76	76	71	74	74	74	68	57	52	48	43	37	31	18	11
62	0	74	92	96	92	80	85	87	85	85	68	58	54	51	45	35	12	2
63	0	59	74	74	79	81	81	81	80	79	74	70	70	70	68	67	63	55
64	0	48	59	62	62	62	62	62	62	58	55	49	46	46	37	33	33	27
65	0	44	44	43	43	36	31	31	27	27	35	30	30	25	25	25	25	19
66	0	81	80	76	76	74	74	74	71	68	70	69	64	57	51	51	47	37
67	0	40	49	55	70	75	80	84	88	93	93	93	86	82	80	77	75	56
68	0	55	65	67	67	67	67	67	67	67	65	59	55	51	46	24	5	0
69	0	57	58	63	64	64	63	59	55	55	55	46	42	40	35	18	13	12
70	0	41	69	77	80	85	87	88	90	90	76	63	34	8	2	0	0	0
71	0	30	41	52	56	60	63	67	69	68	66	63	57	54	35	4	0	0
72	0	56	70	82	82	82	81	81	74	74	81	71	70	58	55	54	52	47
73	0	62	65	65	65	65	59	59	59	48	32	32	26	22	22	22	21	21
74	0	19	47	51	48	49	51	42	42	34	20	15	14	9	0	0	0	0

Hyp-Gra 255	Hyp-Gra 270	Hyp-Gra 285	Hyp-Gra 300	Gra 0	Gra 15	Gra 30	Gra 45	Gra 60	Gra 75	Gra 90	Gra 105	Gra 120	Gra 135	Gra 150	Gra 165	Gra 180	Gra 195	Gra 210	Gra 225	Gra 240	Gra 255
7	5	4	0	14	14	14	14	14	12	10	0	0	0	0	0	0	0	0	0	0	0
0	0	0	0	0	0	0	0	0	0	0	0	0	0	0	0	0	0	0	0	0	0
0	0	0	0	36	36	36	29	27	0	0	0	0	0	0	0	0	0	0	0	0	0
2	1	0	0	0	0	0	0	0	4	4	5	5	4	0	0	0	0	0	0	0	0
0	0	0	0	96	89	92	85	87	89	79	70	66	56	54	44	23	11	4	2	0	0
0	0	0	0	13	11	9	8	7	4	6	5	7	0	0	0	0	0	0	0	0	0
6	6	7	7	7	7	7	7	6	7	7	8	8	6	7	6	6	7	7	7	6	6
0	0	0	0	52	3	3	2	2	2	1	1	0	0	0	0	0	0	0	0	0	0
4	1	2	1	0	1	0	1	0	0	0	1	0	0	0	0	0	0	0	0	0	0
0	2	2	0	100	100	100	100	100	100	95	92	85	69	52	44	35	33	20	7	7	3
0	0	0	0	1	0	0	0	0	0	0	0	0	0	0	0	0	0	0	0	0	0
0	0	0	0	0	1	1	2	0	0	0	0	0	0	0	0	0	0	0	0	0	0
0	0	0	0	27	25	24	20	17	5	1	0	0	0	0	0	0	0	0	0	0	0
0	0	0	0	3	3	9	16	29	29	26	25	15	12	7	0	0	0	0	0	0	0
0	0	0	0	8	3	3	0	0	0	0	0	0	0	0	0	0	0	0	0	0	0
0	0	0	0	0	0	0	0	0	0	0	0	0	0	0	0	0	0	0	0	0	0
9	7	5	5	0	0	0	0	0	0	0	0	0	0	0	0	0	0	0	0	0	0
0	0	0	0	1	30	27	25	23	20	16	7	0	0	0	0	0	0	0	0	0	0
0	0	0	0	2	0	0	0	0	0	0	0	0	0	0	0	0	0	0	0	0	0
0	0	0	0	4	11	12	13	9	5	3	3	0	0	0	0	0	0	0	0	0	0
12	10	7	2	2	2	5	6	10	9	10	8	6	5	3	0	0	0	0	0	0	0
20	14	7	3	49	60	62	63	64	65	55	46	45	27	16	9	0	0	0	0	0	0
0	0	0	0	20	7	3	0	0	0	0	0	0	0	0	0	0	0	0	0	0	0
6	3	0	0	5	5	6	5	4	2	0	0	0	0	0	0	0	0	0	0	0	0
0	0	0	0	1	0	1	2	2	2	1	0	0	0	0	0	0	0	0	0	0	0
0	0	0	0	25	24	21	19	14	2	0	0	0	0	0	0	0	0	0	0	0	0
27	21	11	2	0	0	0	0	0	0	0	0	0	0	0	0	0	0	0	0	0	0
25	22	0	0	0	63	70	74	74	62	62	52	52	52	10	10	5	0	0	0	0	0
0	0	0	0	0	0	0	0	0	0	0	0	0	0	0	0	0	0	0	0	0	0
0	0	0	0	0	0	0	0	0	0	0	0	0	0	0	0	0	0	0	0	0	0
0	0	0	0	12	12	12	12	12	12	11	2	0	0	0	0	0	0	0	0	0	0
8	4	0	0	0	0	0	0	0	0	0	0	0	0	0	0	0	0	0	0	0	0
3	3	0	0	2	2	5	5	4	7	4	3	3	2	1	0	0	0	0	0	0	0
41	40	40	41	0	4	5	5	7	7	7	8	8	5	2	0	0	0	0	0	0	0
8	4	0	0	51	56	60	66	76	80	80	80	65	56	34	15	0	0	0	0	0	0
1	0	0	0	4	0	0	0	0	0	0	0	0	0	0	0	0	0	0	0	0	0
0	0	0	0	0	0	0	0	0	0	0	0	0	0	0	0	0	0	0	0	0	0
51	29	27	27	0	0	0	0	0	0	0	0	0	0	0	0	0	0	0	0	0	0
8	5	5	5	27	1	0	0	0	0	0	0	0	0	0	0	0	0	0	0	0	0
11	7	0	0	0	0	0	0	0	0	0	0	0	0	0	0	0	0	0	0	0	0
10	7	4	2	2	0	0	0	0	0	0	0	0	0	0	0	0	0	0	0	0	0
37	35	35	31	8	3	0	0	0	0	0	0	0	0	0	0	0	0	0	0	0	0
4	2	2	1	54	47	19	1	0	0	0	0	0	0	0	0	0	0	0	0	0	0
24	20	18	14	54	38	21	13	0	0	0	0	0	0	0	0	0	0	0	0	0	0
0	0	0	0	0	0	0	0	0	0	0	0	0	0	0	0	0	0	0	0	0	0
24	15	10	8	0	0	0	0	0	0	0	0	0	0	0	0	0	0	0	0	0	0
4	4	0	0	33	14	9	4	0	0	0	0	0	0	0	0	0	0	0	0	0	0
2	0	0	0	74	80	80	84	84	80	80	78	63	43	43	40	23	20	8	0	0	0
52	51	47	46	32	12	11	7	2	2	0	0	0	0	0	0	0	0	0	0	0	0
19	10	11	4	0	0	0	0	0	0	0	0	0	0	0	0	0	0	0	0	0	0
18	13	13	12	25	21	20	14	13	9	8	4	2	1	0	0	0	0	0	0	0	0
30	30	27	29	0	0	0	0	0	0	0	0	0	0	0	0	0	0	0	0	0	0
46	41	27	19	14	14	14	8	4	0	0	0	0	0	0	0	0	0	0	0	0	0
0	0	0	0	0	0	0	0	0	0	0	0	0	0	0	0	0	0	0	0	0	0
5	0	0	0	0	0	0	0	0	0	0	0	0	0	0	0	0	0	0	0	0	0
0	0	0	0	0	0	0	0	0	0	0	0	0	0	0	0	0	0	0	0	0	0
0	0	0	0	3	0	0	0	0	0	0	0	0	0	0	0	0	0	0	0	0	0
45	31	29	15	18	18	18	18	13	13	11	4	3	0	0	0	0	0	0	0	0	0
20	20	8	4	0	0	0	0	0	0	0	0	0	0	0	0	0	0	0	0	0	0
0	0	0	0	0	0	0	0	0	0	0	0	0	0	0	0	0	0	0	0	0	0

Gra 270	Gra 285	Gra 300	Hyp BL	Hyp 0	Hyp 15	Hyp 30	Hyp 45	Hyp 60	Hyp 75	Hyp 90	Hyp 105	Hyp 120	Hyp 135	Hyp 150	Hyp 165	Hyp 180	Hyp 195	Hyp 210	Hyp 225	Hyp 240	Hyp 255	Hyp 270	Hyp 285
0	0	0	0	79	86	89	92	92	92	92	92	88	74	46	37	28	20	14	13	10	9	6	6
0	0	0	0	74	79	84	88	92	95	95	90	88	74	66	49	41	31	23	14	0	0	0	0
0	0	0	0	23	26	32	39	39	41	41	42	42	35	35	35	29	19	13	7	0	0	0	0
0	0	0	0	70	72	74	70	72	73	75	76	79	59	58	58	38	28	15	9	4	3	2	0
0	0	0	0	91	79	92	93	80	80	72	72	68	69	60	51	32	20	5	4	0	0	0	0
0	0	0	0	17	28	32	24	19	14	15	3	0	0	0	0	0	0	0	0	0	0	0	0
5	6	6	0	6	6	10	10	11	9	10	11	11	13	12	11	11	10	10	10	10	10	10	11
0	0	0	0	85	84	84	92	82	71	45	22	7	3	3	3	3	2	2	0	0	0	0	0
0	0	0	0	82	85	84	79	60	60	43	37	31	41	30	24	17	12	7	3	0	0	0	0
0	0	0	0	93	98	98	98	98	98	98	98	98	42	39	40	22	10	9	7	3	3	2	0
0	0	0	0	31	37	38	39	38	33	12	2	0	0	0	0	0	0	0	0	0	0	0	0
0	0	0	0	16	19	25	30	31	1	1	2	2	0	0	0	0	0	0	0	0	0	0	0
0	0	0	0	57	57	57	58	55	55	53	50	45	37	32	26	16	6	5	1	0	0	0	0
0	0	0	0	8	12	17	22	23	23	21	19	18	12	10	6	4	3	0	0	0	0	0	0
0	0	0	0	49	61	72	77	76	76	71	64	55	46	43	44	37	36	35	30	25	23	22	22
0	0	0	0	69	65	62	58	43	39	29	25	16	10	4	1	0	0	0	0	0	0	0	0
0	0	0	0	12	20	26	31	36	39	42	44	47	31	34	35	36	36	37	37	37	35	29	21
0	0	0	0	51	59	61	62	64	64	59	44	35	27	23	22	14	8	4	1	1	0	0	0
0	0	0	0	31	54	62	67	70	72	74	74	74	70	68	65	60	35	15	5	3	0	0	0
0	0	0	0	16	17	20	18	18	20	21	19	23	20	18	15	9	5	2	1	0	0	0	0
0	0	0	0	11	50	58	61	72	71	71	70	66	57	51	47	43	40	37	34	24	22	19	17
0	0	0	0	83	83	88	93	94	91	93	87	86	94	88	77	54	39	37	27	17	16	6	2
0	0	0	0	58	54	52	53	52	46	45	42	39	36	28	22	17	15	10	7	3	3	0	0
0	0	0	0	16	23	30	31	38	38	40	32	22	27	25	24	20	17	15	12	8	4	3	1
0	0	0	0	52	62	64	64	64	63	60	55	49	35	26	16	14	13	11	4	2	0	0	0
0	0	0	0	47	50	52	54	59	60	60	64	65	58	48	45	35	23	10	9	4	2	0	0
0	0	0	0	51	73	74	69	74	76	78	70	54	47	40	31	29	29	23	18	17	16	11	2
0	0	0	0	67	78	92	91	93	93	93	93	93	92	92	69	69	58	55	55	44	37	30	22
0	0	0	0	24	26	32	32	32	30	35	34	36	22	18	8	0	0	0	0	0	0	0	0
0	0	0	0	15	52	38	44	41	41	41	34	34	30	23	15	9	3	1	0	0	0	0	0
0	0	0	0	12	18	7	7	8	8	8	8	7	7	5	3	0	0	0	0	0	0	0	0
0	0	0	0	75	69	69	64	63	57	49	40	36	33	29	26	23	19	16	8	0	0	0	0
0	0	0	0	27	37	65	68	73	74	73	74	71	69	68	65	56	51	45	41	38	35	27	12
0	0	0	0	73	84	91	95	96	98	98	98	96	96	98	96	96	95	98	92	92	91	63	45
0	0	0	0	71	75	77	79	82	85	85	85	81	79	77	74	35	30	21	18	12	8	5	0
0	0	0	0	92	84	95	89	85	85	85	85	81	75	76	69	65	63	56	21	8	7	0	0
0	0	0	0	9	19	24	22	24	19	19	19	12	8	0	0	0	0	0	0	0	0	0	0
0	0	0	0	96	97	99	100	100	100	100	100	100	100	93	93	93	93	92	89	58	58	58	58
0	0	0	0	66	73	74	74	74	74	74	74	74	73	71	71	70	63	62	52	36	24	12	8
0	0	0	0	86	75	84	91	81	82	82	82	82	78	85	85	86	79	76	71	67	23	9	2
0	0	0	0	33	55	68	69	71	74	65	55	54	49	43	37	27	27	13	11	8	8	3	2
0	0	0	0	54	53	55	55	51	48	48	44	45	43	36	34	34	30	24	22	15	10	7	5
0	0	0	0	34	74	77	75	74	74	74	69	67	65	60	52	47	26	26	19	15	8	2	0
0	0	0	0	78	78	76	73	73	74	68	65	63	63	47	44	40	34	30	29	24	23	21	21
0	0	0	0	21	26	30	30	29	27	27	27	22	19	14	8	3	3	0	0	0	0	0	0
0	0	0	0	5	13	27	31	58	55	56	70	79	81	53	46	43	42	40	37	33	29	22	12
0	0	0	0	66	64	64	64	62	64	65	65	57	57	56	53	45	37	31	19	10	4	3	0
0	0	0	0	75	84	92	87	84	84	87	85	85	66	56	54	46	41	30	11	2	2	0	0
0	0	0	0	36	43	43	47	49	49	49	46	46	38	36	36	36	34	32	29	25	23	21	21
0	0	0	0	49	57	59	59	59	59	59	59	57	57	51	47	47	40	35	35	27	20	12	12
0	0	0	0	57	59	59	59	56	54	48	48	46	35	35	35	35	35	36	33	35	35	35	35
0	0	0	0	81	81	78	79	76	76	76	74	71	67	67	66	58	51	51	48	41	35	35	33
0	0	0	0	37	48	47	65	70	75	79	82	89	95	95	89	87	86	84	81	57	46	40	27
0	0	0	0	57	63	66	66	66	66	66	66	66	66	58	55	49	44	40	37	32	15	12	5
0	0	0	0	63	65	68	70	69	69	66	66	66	66	62	65	62	57	29	24	14	5	0	0
0	0	0	0	40	57	47	41	18	1	0	0	0	0	0	0	0	0	0	0	0	0	0	0
0	0	0	0	36	44	54	58	62	63	60	59	55	52	48	45	42	22	3	0	0	0	0	0
0	0	0	0	67	80	87	87	88	88	88	87	87	84	81	81	74	68	66	60	55	51	38	33
0	0	0	0	56	64	64	64	64	60	59	59	52	36	36	30	30	25	22	10	8	7	7	3
0	0	0	0	27	48	52	45	47	47	35	35	25	16	12	11	8	0	0	0	0	0	0	0

Hyp 300	Hyp-hyp 0	Hyp-hyp 15	Hyp-hyp 30	Hyp-hyp 45	Hyp-hyp 60	Hyp-hyp 75	Hyp-hyp 90	Hyp-hyp 105	yp-hyp 120	Hyp-hyp 135	Hyp-hyp 150	Hyp-hyp 165	Hyp-hyp 180	Hyp-hyp 195	Hyp-hyp 210	Hyp-hyp 225	Hyp-hyp 240	Hyp-hyp 255	Hyp-hyp 270
0	38	63	63	68	72	83	87	90	90	76	73	63	51	45	28	19	10	8	3
0	47	59	62	67	69	72	74	71	78	69	68	56	36	29	26	12	8	4	2
0	0	43	45	35	44	0	0	0	0	0	0	0	0	0	0	0	0	0	0
0	16	21	24	27	27	30	33	36	38	32	27	25	12	7	7	4	1	0	0
0	65	86	86	85	81	78	70	64	63	63	62	54	44	22	3	6	6	1	0
0	24	37	40	43	41	34	32	25	20	6	5	0	0	0	0	0	0	0	0
12	28	29	39	41	42	41	41	41	35	33	33	33	33	30	30	29	27	26	24
0	50	70	47	25	3	3	3	3	0	0	0	0	0	0	0	0	0	0	0
0	54	50	47	34	31	5	2	0	0	0	0	0	0	0	0	0	0	0	0
0	100	100	100	100	100	100	88	75	75	35	32	30	27	28	16	0	0	0	0
0	48	54	57	47	52	43	17	7	2	0	0	0	0	0	0	0	0	0	0
0	49	56	53	55	58	55	50	44	36	9	7	3	2	1	0	0	0	0	0
0	45	45	45	46	42	32	26	25	18	10	6	1	0	0	0	0	0	0	0
0	3	5	5	14	28	20	17	16	10	8	4	3	2	0	0	0	0	0	0
22	8	4	4	0	0	0	0	0	0	0	0	0	0	0	0	0	0	0	0
0	73	76	77	77	74	67	54	41	35	24	19	16	14	13	11	3	2	1	0
9	7	8	8	10	11	12	12	13	14	11	10	10	9	9	7	5	6	5	2
0	3	47	55	62	64	63	61	52	35	26	14	5	1	1	0	0	0	0	0
0	2	36	41	42	42	42	39	37	33	36	15	5	3	2	0	0	0	0	0
0	4	13	27	32	27	29	26	26	25	16	8	5	1	0	0	0	0	0	0
13	13	21	26	32	37	37	40	42	40	34	34	34	33	30	30	29	27	26	24
1	61	89	94	92	90	89	79	75	59	44	24	11	4	0	0	0	0	0	0
0	34	38	44	41	44	42	40	38	34	27	21	20	14	11	8	4	2	0	0
0	10	17	23	26	23	20	16	9	5	0	0	0	0	0	0	0	0	0	0
0	23	26	31	30	29	28	27	24	24	22	16	9	4	0	0	0	0	0	0
0	35	57	59	61	69	59	46	32	24	17	9	7	3	0	0	0	0	0	0
3	79	80	82	83	83	83	80	77	52	49	38	32	26	13	5	1	0	0	0
0	0	0	0	0	0	0	0	0	0	0	0	0	0	0	0	0	0	0	0
0	0	12	12	12	10	10	9	12	11	10	4	1	0	0	0	0	0	0	0
0	0	4	19	21	30	32	33	30	31	30	22	18	18	15	14	11	8	2	1
0	20	26	26	26	30	30	32	35	35	23	19	13	9	7	3	0	0	0	0
0	70	71	73	68	65	47	41	22	12	3	0	0	0	0	0	0	0	0	0
3	20	33	41	57	63	65	68	68	68	52	47	34	24	12	10	3	2	1	0
45	3	21	21	21	21	21	26	27	29	25	26	19	11	12	12	9	9	4	0
0	0	0	0	0	0	0	0	0	0	0	0	0	0	0	0	0	0	0	0
0	96	96	92	88	78	58	58	40	27	8	2	0	0	0	0	0	0	0	0
0	4	8	8	10	7	13	12	9	8	4	0	0	0	0	0	0	0	0	0
47	7	58	100	100	100	100	100	100	95	75	71	49	49	24	15	0	0	0	0
8	48	51	51	51	51	51	51	51	49	47	43	21	8	0	0	0	0	0	0
2	92	92	87	87	86	90	95	95	87	65	42	22	13	7	0	0	0	0	0
1	48	48	52	52	49	48	44	41	31	11	8	4	2	2	1	0	0	0	0
3	53	53	48	48	45	43	43	40	36	29	25	21	18	13	11	7	4	0	0
0	82	86	82	73	71	65	53	52	46	34	22	12	5	3	3	0	0	0	0
16	82	80	77	71	66	60	57	48	45	34	30	21	16	4	0	0	0	0	0
0	11	19	25	27	31	33	33	33	32	31	29	25	20	15	12	4	3	2	1
8	0	10	18	23	26	27	30	30	30	37	27	27	27	25	16	9	9	9	3
0	66	59	56	52	47	44	37	27	13	10	0	0	0	0	0	0	0	0	0
0	74	82	82	86	86	84	84	79	55	45	43	40	36	36	18	12	7	0	0
20	51	51	59	66	69	64	53	48	51	35	32	27	23	13	7	0	0	0	0
4	38	43	46	46	46	46	46	43	43	27	27	24	15	15	7	7	0	0	0
35	23	23	30	32	35	34	34	34	31	27	26	25	23	14	8	3	2	0	0
33	78	78	71	71	71	66	66	66	63	65	51	41	30	24	16	7	0	0	0
20	18	33	35	36	49	54	58	66	68	64	75	76	67	60	59	36	25	27	15
3	34	40	42	42	42	42	42	40	40	31	30	26	7	2	0	0	0	0	0
0	64	64	67	70	70	70	65	65	51	44	24	4	0	0	0	0	0	0	0
0	54	80	85	90	95	96	96	90	82	68	60	40	31	23	5	0	0	0	0
0	45	43	33	24	27	23	16	14	8	4	1	0	0	0	0	0	0	0	0
22	87	86	86	79	75	74	71	71	68	66	53	38	37	30	22	20	10	4	0
0	67	62	70	70	54	52	47	45	45	38	27	21	16	9	5	3	0	0	0
0	11	8	16	16	13	7	4	0	0	0	0	0	0	0	0	0	0	0	0

Hyp-hyp 285	Hyp-hyp 300
1	0
2	0
0	0
0	0
0	0
0	0
22	20
0	0
0	0
0	0
0	0
0	0
0	0
0	0
0	0
0	0
0	0
0	0
0	0
0	0
2	0
0	0
0	0
0	0
0	0
0	0
0	0
0	0
0	0
0	0
0	0
0	0
0	0
0	0
0	0
0	0
0	0
0	0
0	0
0	0
0	0
0	0
0	0
0	0
0	0
3	0
0	0
0	0
0	0
0	0
0	0
0	0
7	0
0	0
0	0
0	0
0	0
0	0
0	0
0	0
