# Supplementary material for: Influence of Polymorphisms in the HTR3A and HTR3B Genes on Experimental Pain and the Effect of the 5-HT3 Antagonist Granisetron
Source: PLoS One. 2016 Dec 21;11(12):e0168703. doi: 10.1371/journal.pone.0168703 (PMC5176308; doi:10.1371/journal.pone.0168703)
Supplement: S2 Appendix — (PDF) [file pone.0168703.s002.pdf]

Pat nr	Sex	Sample name	VAS peak 1	VAS dur 1	VAS peak 2	VAS dur 2	Treat effect % peak gra	Treat effect % dur gra	HTR3A	HTR3B
1	F	1001	92	285	14	90	84,7826087	68,42105263	Heterozygous C/T	Heterozygous A/C
2	F	1002	95	225	0	0	100	100	Homozygous C/C	Heterozygous A/C
3	F	1003	42	225	36	60	14,28571429	73,33333333	Homozygous C/C	Homozygous C/C
4	F	1004	79	270	5	135	93,67088608	50	Homozygous C/C	Homozygous A/A
5	F	1005	93	225	92	225	1,075268817	0	Homozygous C/C	Homozygous C/C
9	F	1009	32	105	13	120	59,375	-14,28571429	Homozygous C/C	Homozygous A/A
11	F	1011	13	300	8	300	38,46153846	0	Homozygous C/C	Heterozygous A/C
13	F	1013	92	210	52	105	43,47826087	50	Homozygous C/C	Heterozygous A/C
14	F	1014	85	225	1	105	98,82352941	53,33333333	Homozygous C/C	Heterozygous A/C
15	F	1015	98	270	100	255	-2,040816327	5,555555556	Homozygous C/C	Homozygous A/A
16	M	1016	39	105	1	0	97,43589744	100	Homozygous C/C	Homozygous C/C
19	M	1019	31	120	2	45	93,5483871	62,5	Homozygous T/T	Homozygous A/A
23	M	1023	58	225	27	90	53,44827586	60	Homozygous C/C	Heterozygous A/C
25	M	1025	23	195	29	150	-26,08695652	23,07692308	Homozygous C/C	Heterozygous A/C
26	M	1026	77	300	8	30	89,61038961	90	Homozygous C/C	Heterozygous A/C
27	M	1027	69	165	0	0	100	100	Heterozygous C/T	Heterozygous A/C
29	M	1029	47	300	0	0	100	100	Heterozygous C/T	Homozygous A/A
31	F	1031	64	240	30	105	53,125	56,25	Heterozygous C/T	Homozygous C/C
32	M	1032	74	240	2	0	97,2972973	100	Homozygous C/C	Heterozygous A/C
33	M	1033	23	225	13	105	0	53,33333333	Heterozygous C/T	Heterozygous A/C
35	M	1035	71	300	10	150	85,91549296	50	Heterozygous C/T	Homozygous A/A
36	M	1036	94	300	65	165	30,85106383	45	Homozygous C/C	Homozygous A/A
37	F	1037	58	255	20	30	65,51724138	88,23529412	Heterozygous C/T	Heterozygous A/C
38	F	1038	40	300	6	75	85	75	Homozygous C/C	Homozygous C/C
39	F	1039	64	240	2	90	96,875	62,5	Heterozygous C/T	Heterozygous A/C
40	F	1040	65	255	25	75	61,53846154	70,58823529	Homozygous C/C	Homozygous A/A
41	M	1041	78	300	0	0	100	100	Homozygous C/C	Homozygous A/A
42	F	1042	93	285	74	165	20,43010753	42,10526316	Heterozygous C/T	Homozygous A/A
43	M	1043	35	165	0	0	100	100	Heterozygous C/T	Heterozygous A/C
44	F	1044	52	210	0	0	100	100	Heterozygous C/T	Homozygous A/A
45	M	1045	18	165	12	105	33,33333333	36,36363636	Homozygous C/C	Heterozygous A/C
46	F	1046	75	225	0	0	100	100	Homozygous C/C	Heterozygous A/C
47	F	1047	74	300	7	150	90,54054054	50	Heterozygous C/T	Homozygous A/A
48	F	1048	98	300	8	165	91,83673469	45	Homozygous C/C	Homozygous A/A
49	F	1049	85	270	80	165	5,882352941	38,88888889	Homozygous C/C	Heterozygous A/C
50	F	1050	95	255	4	0	95,78947368	100	Homozygous C/C	Homozygous A/A
51	M	1051	24	135	0	0	100	100	Homozygous C/C	Homozygous C/C
52	M	1052	100	300	0	0	100	100	Homozygous C/C	Homozygous C/C
53	M	1053	74	300	27	15	63,51351351	95	Heterozygous C/T	Heterozygous A/C
54	F	1054	91	300	0	0	100	100	Homozygous C/C	Heterozygous A/C
55	F	1055	71	300	2	0	97,18309859	100	Homozygous C/C	Homozygous A/A
56	M	1056	55	300	8	15	85,45454545	95	Heterozygous C/T	Homozygous C/C
57	F	1057	77	270	54	45	29,87012987	83,33333333	Heterozygous C/T	Homozygous C/C
58	F	1058	78	300	54	45	30,76923077	85	Heterozygous C/T	Heterozygous A/C
59	M	1059	30	195	0	0	100	100	Homozygous C/C	Homozygous A/A
60	M	1060	79	300	0	0	100	100	Heterozygous C/T	Heterozygous A/C
61	F	1061	66	255	33	45	50	82,35294118	Homozygous C/C	Homozygous A/A
62	F	1062	92	255	84	210	8,695652174	17,64705882	Heterozygous C/T	Homozygous A/A
63	M	1063	49	300	32	75	34,69387755	75	Heterozygous C/T	Heterozygous A/C
64	M	1064	59	300	0	0	100	100	Heterozygous C/T	Heterozygous A/C
65	M	1065	59	300	25	135	57,62711864	55	Homozygous C/C	Homozygous A/A
66	M	1066	81	300	0	0	100	100	Homozygous T/T	Heterozygous A/C
67	M	1067	95	300	14	60	85,26315789	80	Heterozygous C/T	Homozygous A/A
68	M	1068	66	300	0	0	100	100	Homozygous C/C	Heterozygous A/C
69	F	1069	70	255	0	0	100	100	Homozygous C/C	Homozygous A/A
70	F	1070	52	75	0	0	100	100	Homozygous C/C	Homozygous C/C
71	M	1071	63	240	3	0	95,23809524	100	Homozygous C/C	Homozygous A/A
72	M	1072	88	300	18	120	79,54545455	60	Heterozygous C/T	Homozygous A/A
73	M	1073	64	285	0	0	100	100	Homozygous C/C	Heterozygous A/C
74	M	1074	52	180	0	0	100	100	Homozygous C/C	Homozygous C/C
