# Supplementary material for: Influence of Polymorphisms in the HTR3A and HTR3B Genes on Experimental Pain and the Effect of the 5-HT3 Antagonist Granisetron
Source: PLoS One. 2016 Dec 21;11(12):e0168703. doi: 10.1371/journal.pone.0168703 (PMC5176308; doi:10.1371/journal.pone.0168703)
Supplement: S3 Appendix — (PDF) [file pone.0168703.s003.pdf]

Pat nr	Sex	Area 1	Area 2	Normalized	HTR3A	HTR3B
1	F	368	0	100	Heterozygous C/T	Heterozygous A/C
37	F	149	44	70,46979866	Heterozygous C/T	Heterozygous A/C
39	F	68	50	26,47058824	Heterozygous C/T	Heterozygous A/C
58	F	45	44	2,222222222	Heterozygous C/T	Heterozygous A/C
2	F	189	0	100	Homozygous C/C	Heterozygous A/C
11	F	14	16	-14,28571429	Homozygous C/C	Heterozygous A/C
13	F	86	7	91,86046512	Homozygous C/C	Heterozygous A/C
14	F	10	1	90	Homozygous C/C	Heterozygous A/C
46	F	12	0	100	Homozygous C/C	Heterozygous A/C
49	F	54	50	7,407407407	Homozygous C/C	Heterozygous A/C
54	F	18	0	100	Homozygous C/C	Heterozygous A/C
27	M	45	0	100	Heterozygous C/T	Heterozygous A/C
33	M	57	28	50,87719298	Heterozygous C/T	Heterozygous A/C
43	M	17	0	100	Heterozygous C/T	Heterozygous A/C
53	M	183	0	100	Heterozygous C/T	Heterozygous A/C
60	M	14	0	100	Heterozygous C/T	Heterozygous A/C
63	M	17	0	100	Heterozygous C/T	Heterozygous A/C
64	M	4	0	100	Heterozygous C/T	Heterozygous A/C
23	M	29	27	6,896551724	Homozygous C/C	Heterozygous A/C
25	M	14	15	-7,142857143	Homozygous C/C	Heterozygous A/C
26	M	13	0	100	Homozygous C/C	Heterozygous A/C
32	M	24	0	100	Homozygous C/C	Heterozygous A/C
45	M	14	15	-7,142857143	Homozygous C/C	Heterozygous A/C
68	M	32	0	100	Homozygous C/C	Heterozygous A/C
73	M	141	0	100	Homozygous C/C	Heterozygous A/C
66	M	48	0	100	Homozygous T/T	Heterozygous A/C
42	F	0	149		Heterozygous C/T	Homozygous A/A
44	F	34	0	100	Heterozygous C/T	Homozygous A/A
47	F	70	4	94,28571429	Heterozygous C/T	Homozygous A/A
62	F	50	28	44	Heterozygous C/T	Homozygous A/A
4	F	20	0	100	Homozygous C/C	Homozygous A/A
9	F	42	10	76,19047619	Homozygous C/C	Homozygous A/A
15	F	15	16	-6,666666667	Homozygous C/C	Homozygous A/A
40	F	42	52	-23,80952381	Homozygous C/C	Homozygous A/A
48	F	120	17	85,83333333	Homozygous C/C	Homozygous A/A
50	F	30	0	100	Homozygous C/C	Homozygous A/A
55	F	169	60	64,49704142	Homozygous C/C	Homozygous A/A
61	F	53	0	100	Homozygous C/C	Homozygous A/A
69	F	108	0	100	Homozygous C/C	Homozygous A/A
29	M	19	0	100	Heterozygous C/T	Homozygous A/A
35	M	87	0	100	Heterozygous C/T	Homozygous A/A
67	M	96	9	90,625	Heterozygous C/T	Homozygous A/A
72	M	202	4	98,01980198	Heterozygous C/T	Homozygous A/A
36	M	26	29	-11,53846154	Homozygous C/C	Homozygous A/A
41	M	20	2	90	Homozygous C/C	Homozygous A/A
59	M	29	0	100	Homozygous C/C	Homozygous A/A
65	M	5	0	100	Homozygous C/C	Homozygous A/A
71	M	19	0	100	Homozygous C/C	Homozygous A/A
19	M	24	0	100	Homozygous T/T	Homozygous A/A
31	F	46	9	80,43478261	Heterozygous C/T	Homozygous C/C
57	F	10	1	90	Heterozygous C/T	Homozygous C/C
3	F	55	43	21,81818182	Homozygous C/C	Homozygous C/C
5	F	81	83	-2,469135802	Homozygous C/C	Homozygous C/C
38	F	159	13	91,82389937	Homozygous C/C	Homozygous C/C
70	F	148	0	100	Homozygous C/C	Homozygous C/C
56	M	80	0	100	Heterozygous C/T	Homozygous C/C
16	M	12	0	100	Homozygous C/C	Homozygous C/C
51	M	18	0	100	Homozygous C/C	Homozygous C/C
52	M	45	0	100	Homozygous C/C	Homozygous C/C
74	M	15	0	100	Homozygous C/C	Homozygous C/C
