# Supplementary material for: Influence of Polymorphisms in the HTR3A and HTR3B Genes on Experimental Pain and the Effect of the 5-HT3 Antagonist Granisetron
Source: PLoS One. 2016 Dec 21;11(12):e0168703. doi: 10.1371/journal.pone.0168703 (PMC5176308; doi:10.1371/journal.pone.0168703)
Supplement: S4 Appendix — (PDF) [file pone.0168703.s004.pdf]

Pat	PPT hyp BL	PPT hyp 5 min	PPT hyp 10 min	PPT hyp 15 min	PPT hyp 20 min	PPT hyp 25 min	PPT hyp 30 min	PPT2 hyp BL	PPT2 hyp 5 min	PPT2 hyp 10 min	PPT2 hyp 15 min	PPT2 hyp 20 min	PPT2 hyp 25 min	PPT2 hyp 30 min
1	233	227	286,5	186,5	131	101,5	182	182	179,5	127	158	214	195	225
2	190	182,5	190	174,5	200,5	190	163,5	163,5	216	138	242	155,5	156	137
3	252,333	377,5	285	315,5	288	401	423,5	423,5	342	263	302	354,5	248,5	314,5
4	210,333	280	207,5	215,5	203	206	200	200	176	218	185,5	182	198	197,5
5	237	235,5	247	211,5	227,5	203	188,5	188,5	224	209	207	207	187	195,5
9	197,666	193,5	216	195	191,5	179	178	178	181,5	218,5	169	183	165,5	159
11	288	264	286	286	311	305	319	319	323	337	304	374	376	331
13	145,333	140,5	144,5	165,5	145	160	127	127	186,5	152	155	181	172	158,5
14	171	190	181	205,5	211,5	202,5	188	188	229,5	215,5	273,5	231,5	195,5	164,5
15	179,333	221,5	183,5	138,5	96,5	167,5	139	149	144,5	151,5	177,5	168,5	148	153,5
16	310,333	303,5	269,5	271	275	244,5	263	263	250	299	318,5	277,5	225	265,5
19	228,333	323,5	348,5	264,5	299	289	318,5	318,5	378	309	343,5	338,5	378	328,5
23	252,333	221	267,5	246,5	225,5	211	193	193	237	249	205	237	228,5	220
25	267,333	209,5	184	183	205	197,5	187,5	187,5	243	200,5	206	210	244,5	193,5
26	252,333	226	251,5	231,5	242	167	187	187	237	187	167	233	212	208,5
27	189,666	126	135	105,5	134,5	156,5	146	146	158,5	162	173	155	178,5	166
29	172	115,5	146	150	154,5	139,5	141	141	149,5	183	152	155	163	167
31	134	154		128			130	130	153		138,5			137
32	154	151		168,5			199,5	199,5	224		206			205
33	202,667	199,5		219			188,5	188,5	227,5		196,5			201
35	240,333	239,5		264			257	257	314,5		206,5			277,5
36	151,667	110,5		112			115,5	115,5	107		120			142
37	136,333	138		121			108	108	83,5		134,5			105,5
38	153,667	202		237			279,5	279,5	238		272			290
39	183,667	178		181			130	130	166,5		158			172
40	202,333	180,5		142			190,5	190,5	141,5		150			139
41	132,333	111,5		72,5			56	56	68		71,5			86
42	216,333	225,5	261	202	200,5	221	253	253	190,5	181	137,5	147	137,5	181
43	428,667	426,5	443,5	510	444,5	484	547,5	547,5	496	501	512,5	542	561	542
44	248,667	295,5	262	250,5	281,5	283	251	251	243	218	265	297	288	264,5
45	314,333	246,5	238	310,5	254,5	297	338	338	371	300,5	291,5	328,5	323	312
46	273,667	301,5	307	291,5	353,5	293	276	276	272	296,5	294	323	333	298
47	159,333	115,5	142	131	155	177	172,667	172,667	114	136	138	153,5	154	150
48	151	93	125	149	142	132	142	142	58	89	92,5	84,5	77,5	82
49	285	391	380,5	306,5	379	141,5	400,5	400,5	373,5	371,5	330,5	333	330,5	336,5
50	191	179,5	162	188	169,5	211,5	203	203	163	146,5	225	257	269	287
51	223	266,5	241,5	254	285	247	238,5	238,5	238	250	228	209,5	258,5	227,5
52	318,667	229	204,5	265	271,5	252,5	249	249	252	232	270	246,5	241	296,5
53	257	206	280,5	275	244	250,5	263	236	236	265,5	270	256	284,5	277
54	288,667	275	260	207	217	198	227,5	227,5	171,5	181	207,5	184,5	189,5	170
55	157,667	127,5	176,5	173,5	127,5	104	141,5	141,5	161,5	172	545	188	152,5	176
56	279,333	174	181,5	186	220	200,5	233,5	233,5	246	282,5	288	249,5	283	273,5
57	242,667	252	252	154,5	195	235,5	249	249	255	199,5	198,5	158	187	219
58	228,333	232	224,5	208	198,5	205,5	197	197	188	221,5	238	255,5	240,5	243,5
59	408,667	326,5	295,5	308	351,5	335	331,5	331,5	256	297	300	328,5	303,5	299,5
60	376	374,5	327,5	371	379,5	373,5	636,5	363,5	430	439	400,5	382,5	407,5	384,5
61	239,667	245,5	230	213	221,5	259	224,5	224,5	245,5	203,5	205,5	209	207,5	212
62	209,333	200	182	161	200	182	192	192	206	183,5	148	141	149	139,5
63	266,333	302	286	362	307,5	380	361,5	361,5	409,5	510,5	347	312,5	307,5	331
64	192,667	223	213,5	233,5	233,5	195,5	226,5	226,5	208,5	173,5	261,5	223	256	227,5
65	446	341,5	365	385,5	422,5	363,5	351,5	351,5	410,5	371	377	369	418,5	392
66	425,333	299,5	308	340	329	311,5	338,5	338,5	298	361,5	327,5	347,5	381,5	346
67	323,333	262,5	296,5	296	335	279,5	347	347	309	336,5	336,5	282	285	255
68	265,667	266	254	247	290	284,5	279,5	279,5	269	228,5	274	284	326,5	317,5
69	138,333	90,5	74	79	90,5	32,5	94,5	94,5	88,5	96	103	85	102,5	99
70	162,333	182	150	167,5	180	177,5	165,5	165,5	219,5	201,5	206	173	154	172,5
71	316,333	302,5	314	330	346	356,5	320	320	323,5	303,5	306,5	305	293	245
72	251,667	189	187	236	252	232	252	252	225,5	219,5	279	259	269	310,5
73	247	242	245	314,5	290	220,5	275,5	275,5	258	227	287,5	292,5	296	284
74	284,333	219,5	217,5	218	231,5	229,5	179,5	179,5	159	155	205,5	221	175,5	241

Pat	NaCl hyp BL	NaCl hyp 5 min	NaCl hyp 10 min	NaCl hyp 15 min	NaCl hyp 20 min	NaCl hyp 25 min	NaCl hyp 30 min	NaCl hyp BL	NaCl hyp 5 min	NaCl hyp 10 min	NaCl hyp 15 min	NaCl hyp 20 min	NaCl hyp 25 min	NaCl hyp 30 min
1	144,333	160,5	148	157,5	108	130,5	101	101	186	214,5	201,5	167,5	202	204
2	221,667	219	218	193,5	239	209	184	184	219,5	203	170,5	184,5	195,5	196
3	248,333	376,5	274,5	254,5	322	330,5	442	442	287	319	267	360	235	223,5
4	215,333	231	220	204,5	194,5	212	168	168	203	189,5	197	222	171,5	178,5
5	243,333	199,5	201,5	197,5	180,5	174	182,5	182,5	164	196,5	180	175	150	155,5
9	240	223	237	208,5	212	202	222	222	218	234,5	215,5	168	186,5	169
11	308,667	298	283	259	332	340	348	348	336	321	336	40	387	348
13	158,667	145	146,5	138	125,5	138,5	155,5	155,5	157	141	134,5	133,5	138	144,5
14	129	139,5	173,5	156	122,5	127	168,5	168,5	185	197	254	232,5	206	180
15	148,667	125	138,5	99	131	150	122	122	67	103	132,5	132,5	108,5	163,5
16	330,333	325,5	316,5	318,5	366,5	354,5	335,5	335,5	283,5	336	365,5	281,5	277	321,5
19	270,333	387,5	338,5	328,5	304	402,5	431,5	431,5	348	372,5	378	431,5	412	416,5
23	252,333	242,5	242	225	213,5	231,5	229	229	194,5	227	208,5	199	229,5	207
25	250	203,5	195,5	196,5	186,5	214,5	191,5	191,5	196	200,5	197	166,5	190,5	186,5
26	220,667	218,5	256,5	191,5	295	137	218	218	263	216,5	159	209,5	188	191
27	170	174,5	116,5	74	113	162,5	131	131	114,5	155	160,5	139	127,5	151,5
29	258	199,5	219,5	214	183,5	162,5	203	203	155	212	175	192	185,5	130
31	142	167,5		167,5			161,5	161,5	153,5		160			149,5
32	184,333	197		158			198	198	191		193			220
33	214	227		205			218	218	240		277			221
35	236,667	220,5		226			254,5	254,5	260,5		208			249,5
36	151,333	131		134			142,5	142,5	134,5		153,5			152
37	154	150		134,5			124	124	122,5		92,5			89
38	179,333	262		224,5			240,5	240,5	222,5		229			255,5
39	173,667	171,5		195,5			181	181	175		188,5			174,5
40	173,333	150		166			186	186	150		157			153,5
41	109,333	90,5		85			101	101	89,5		85,5			84,5
42	260,333	178,5	217	197	179,5	154	201	201	122	110	124	149	139	130
43	477,667	494,5	528,5	537,5	381	560	539	539	453,5	465	474	419,5	418,5	440,5
44	218	174,5	218	228,5	233,5	239,5	210,5	210,5	153,5	199,5	216,5	232	203,5	203
45	268,667	168,5	184	242,5	214,5	254	277	277	181,5	201,5	221,5	242,5	242	240,5
46	233,333	256,5	242	236,5	237,5	223,5	230	230	219,5	225,5	204,5	207	215,5	230,5
47	169,333	128	158	157	164,5	191,5	179	179	134,5	181,5	203,5	170	187	184,5
48	179	94,5	106,5	106,5	120,5	125	112,5	112,5	45,5	89	69	87,5	100,5	68
49	423,333	293	290,5	282,5	273	299,5	289	289	313,5	309	302,5	263,5	247	249
50	239,333	177,5	202,5	1875	197	221,5	192,5	192,5	99,5	124,5	163,5	212	227	231
51	195,667	215,5	130	236	265	237,5	227	227	219	228,5	228,667	281,5	240,5	250
52	272,333	202,5	184,5	249	243	206,5	166,5	166,5	236,5	205	250,5	229,5	253	241,5
53	263	208,5	283,5	260	267,5	249,5	266	266	194,5	252	274,5	281	289	318
54	301,667	131,5	192,5	178	221,5	192	219,5	219,5	179,5	139	178	136,5	92	172
55	185,333	107,5	141	198	208,5	210	205	205	251,5	234,5	214,5	206	198,5	211
56	306,333	273,5	217	226	233,5	261	274,5	274,5	229,5	294,5	289,5	273,5	231	253
57	194	215	185	212	206	190,5	232	232	231	223,5	212	148	175	204
58	209	241,5	178,5	162,5	184,5	166	175,5	175,5	179,5	230	202	230,5	223	236
59	351,333	376	371	325,5	309	271	295	295	280	264	294	284	322	233,5
60	422,667	403,5	353	348,5	367,5	335	333	333	396	388	358	362,5	365,5	408,5
61	217	218	202	242,5	195	242,5	211,5	211,5	217	196,5	193,5	181,5	196	198
62	297,333	278	219,5	157	223	187,5	194,5	194,5	225,5	163	170,5	204	160,5	148
63	303	337	342,5	364	320	342	344	344	320,5	302	366,5	360,5	341	361
64	227,667	147	226	195	225	159	210	210	208	141	205,5	175,5	206	208,5
65	470,333	193	312	312	355,5	376	375	375	396,5	357,5	412	318,5	337,5	423,5
66	473	327,5	343,5	344	366,5	331	367	367	315,5	325	343	370	385,5	336,5
67	318	261	268,5	239,5	245	225	263	263	259	263,5	293	261,5	298,5	317
68	244	234	240,5	233,5	264	250	263	263	85	285,5	211	224,5	221	247
69	159	132,5	112,5	131	123,5	127	139	139	56,5	85	104,5	88	111,5	97,5
70	184	196	186	195	179	202,5	205,5	205,5	219	235,5	212,5	222,5	217,5	190,5
71	257,333	300,5	282,5	267,5	264,5	269,5	262	262	332,5	267	271	248	244,5	260
72	280	226,5	200,5	212,5	250,5	253	260,5	260,5	137	157	196	139	155,5	191,5
73	231,667	249	271	292	328,5	261,5	274	274	272,5	296	304,5	301	259,5	284,5
74	263,333	245,5	248,5	210,5	194,5	200,5	177	177	197	257	221,5	236,5	199	225,5
