# Supplementary material for: Influence of Polymorphisms in the HTR3A and HTR3B Genes on Experimental Pain and the Effect of the 5-HT3 Antagonist Granisetron
Source: PLoS One. 2016 Dec 21;11(12):e0168703. doi: 10.1371/journal.pone.0168703 (PMC5176308; doi:10.1371/journal.pone.0168703)
Supplement: S5 Appendix — (PDF) [file pone.0168703.s005.pdf]

Regionala etikprövningsnämnden i Stockholm

Protokoll 2012/2:1

Diarienummer
Föredragande

Utdrag ur protokoll

Diarienummer:
2011/1955-31/2
Föredragande:
Agneta Karsten

Sökande: Karolinska institutet
Behörig företrädare: Kaj Fried
Projekt: Betydelse av stress och vissa genetiska varianter för patogenes, behandling och utfall vid långvarig muskelsmärta
Forskare som genomför projektet: Malin Ernberg

BESLUT

Nämnden godkänner forskningen med påpekande att

1. i rubriken i informationen till försökspersoner och till patienter bör "långvarig muskelsmärta" ändras till "smärta i käkmuskeln" samt
2. två ord i första meningen i annonsen verkas att saknas.

Beslut expedierat till behörig företrädare.
Kopia för kännedom till ansvarig forskare.

Att utdraget överensstämmer med originalet intygar:

Pernilla Asp, adm sekr

PROTOKOLL 2012/2:1
2012-01-11

Sammanträde i Stockholm

Avdelning 2

Ordförande
Ulla Erlandsson

Ledamöter med vetenskaplig kompetens
Anders Björkman vetenskaplig sekreterare *(infektionssjukdomar)*
Kristian Borg *(neurologi)*
Lena Flyckt *(psykiatri)*
Mats Gillberg *(psykofysiologi)*, deltar inte i ärendena 2011/1962, 2011/1976, 2011/1978, 2011/1985, 2011/1986, 2011/1993, 2011/1994 och 2011/1995
Leif Svensson *(kardiologi)*
Agneta Karsten *(odontologi)*
Anna Nilsson *(pediatrik, infektion och immunologi)*, deltar inte i ärendena 2011/1891, 2011/1908 och 2011/1956 pga. jäv.
Anders Thörne *(kirurgi)*
Thomas Sejersen *(neuropediatrik)*, deltar inte i ärendena 2011/1614 och 2011/1719
Claes-Göran Östensson *(medicin, endokrinologi)*, deltar inte i ärende 2011/1986 pga. jäv

Ledamöter som företräder allmänna intressen
Lars Bergstig
Kemo Ceesay
Eva Klingström
Lena Huss
Ingmar Wallén

Administrativ sekreterare
Pernilla Asp

---

§ 1 Ordföranden förklarar sammanträdet öppnat.

§ 2 Den administrativa sekreteraren anmäler att den vetenskaplige sekreteraren sedan föregående möte den 30 november 2011 har fattat beslut i 27 ärenden som avser ändring av ett godkännande.

§ 3 Ansökningar om etisk granskning av forskningsprojekt, **se Bilaga.**

§ 4 Ordföranden förklarar sammanträdet avslutat och meddelar tid för nästa sammanträde.

Ulla Erlandsson
Ordförande

Anders Björkman
Vetenskaplig sekreterare

Adress	Besöksadress	Telefon	Fax	E-post	Hemsida
FE 289<br>171 77 Stockholm	Nobels väg 9<br>Solna	08-524 800 00	08-524 866 99	kansli@stockholm.epn.se	www.epn.se
