# Supplementary material for: Influence of Polymorphisms in the HTR3A and HTR3B Genes on Experimental Pain and the Effect of the 5-HT3 Antagonist Granisetron
Source: PLoS One. 2016 Dec 21;11(12):e0168703. doi: 10.1371/journal.pone.0168703 (PMC5176308; doi:10.1371/journal.pone.0168703)
Supplement: S6 Appendix — (PDF) [file pone.0168703.s006.pdf]

LÄKEMEDELSVERKET
MEDICAL PRODUCTS AGENCY

Beslut

Kliniska prövningar-Licenser
Arzu Günes Granberg/bs

Datum: 2012-02-21

Eu-nr **2011-006206-27**
Dnr 151:2011/96710

Tdl Malin Ernberg
Karolinska Institutet
Inst. för odontologi
Enheten för orofacial smärta och käkfunktion
Box 4064
141 04 Huddinge

Tillstånd till klinisk läkemedelsprövning
Klinisk prövning av Kytril

Ni har ansökt om tillstånd att genomföra en klinisk läkemedelsprövning.

Läkemedelsverket lämnar med stöd av 14 § läkemedelslagen (1992:859) tillstånd att genomföra den kliniska läkemedelsprövningen.

Kommentar, se bilaga.

På Läkemedelsverkets vägnar

Arzu Günes Granberg
Klinisk utredare

Denna beslutshandling är inte underskriven. Detta påverkar inte beslutets giltighet.

Postadress/Postal address: P.O. Box 26, SE-751 03 Uppsala, SWEDEN
Besöksadress/Visiting address: Dag Hammarskjölds väg 42, Uppsala
Telefon/Phone: +46 (0)18 17 46 00 Fax: +46 (0)18 54 85 66
Internet: www.mpa.se E-mail: registrator@mpa.se
